# Opportunities and challenges in involving people with lived experience of inclusion health as co-researchers in palliative and end of life research: a rapid review and thematic synthesis

**DOI:** 10.1186/s40900-023-00436-3

**Published:** 2023-04-20

**Authors:** Jodie Crooks, Kate Flemming, Caroline Shulman, Briony Hudson

**Affiliations:** 1 Research and Policy, Marie Curie, London, UK; 2 Department of Health Sciences, University of York, York, UK; 3 Pathway, London, UK; 4 Healthy London Partnership, London, UK; 5 Marie Curie Palliative Care Research Department, Division of Psychiatry, University College London, London, UK

**Keywords:** Palliative care, Homeless persons, Prisoners, Substance use disorders, Sex work, Co-research, Lived experience, Inclusion health

## Abstract

**Background:**

Co-research is a collaborative approach to research, promoting involvement of individuals with lived experience of a research area as experts by experience. Recently, the importance of co-research within palliative and end of life care (PEoLC) has been highlighted, yet few recommendations exist regarding best practice for involving inclusion health groups (i.e., groups that are socially excluded, typically experiencing multiple disadvantages that contribute to poor health outcomes).

**Aims:**

To identify and synthesise qualitative literature outlining barriers and facilitators for involving four inclusion health groups (individuals with lived experience of: homelessness, substance use disorder, incarceration or exchanging sex for money) in PEoLC research, from the perspectives of both the researchers and individuals with lived experience.

**Methods:**

This report is a rapid review with thematic synthesis methodology. Three electronic databases were searched (2012–30th August 2022). Thematic synthesis was used to generate themes across qualitative studies.

**Results:**

Three qualitative studies were eligible for inclusion. Two involved individuals with lived experience of incarceration, and one lived experience of homelessness. No papers outlined best-practice guidance for co-research; all offered reflections on the co-research process. Challenges for involvement included: facilitating appropriate reimbursement; overcoming stigma; fear of tokenism; pre-conceived views and the emotional burden of research. Successes and benefits included: advanced level of insight, a two-way learning opportunity and relatability of lived experience co-researchers.

**Conclusions:**

This review did not identify any best-practice guidance for co-production of PEoLC research with inclusion health groups. There are few, good quality, qualitative studies offering insight into challenges and facilitators for lived experience co-researcher involvement. Further research and formal policy development is required to produce formal best-practice guidance to support safe, impactful inclusion in PEoLC research.

**Plain English Summary:**

It is important that researchers work together with people who have lived experience of the topic they are researching. Palliative care is specialised medical care for people living with a terminal illness. There is some collaboration between researchers and people with lived experience in palliative and end-of-life care research. However, some groups of people have been excluded. This includes people experiencing homelessness, or people with drug or alcohol addictions. This review aimed to understand what works and what doesn’t work when involving four excluded groups in palliative and end-of-life research. These groups were people experiencing homelessness, those who had spent time in prison, people with drug or alcohol disorders, and people who exchanged sex for money.

This review used a shortened methodology, which allowed it to be done quickly. Three online academic databases (Medline, PubMed, PsychINFO) were searched for research projects: three papers were included in the review. No clear guidance for working with these groups was found. Analysis identified themes across papers. Challenges for collaboration included: appropriate payment methods; overcoming stigma; fear of being talked down to,; assumptions made before meeting people, and the potential of becoming upset. Successes and benefits included: better understanding of the research topic, the opportunity to learn from one another, and how research participants could relate to lived experience co-researchers. There are few, good quality papers, but more research is needed to produce guidance to support safe, impactful collaboration.

**Supplementary Information:**

The online version contains supplementary material available at 10.1186/s40900-023-00436-3.

## Background

Co-research is defined as collaborative research ‘with’ the population of interest, as opposed to conducting research ‘on’ or ‘about’ them [1]. As a blanket-term, co-research encompasses inclusive research methodologies that involve populations throughout the entire process of research conceptualisation and delivery [2]. Co-research advocates for equal value to be placed on the voices and perspectives of individuals with lived experience, by recognising the unique value of an individual’s lived experience [3, 4]. The practice of co-research challenges the asymmetrical power dynamics typically displayed between researchers and participants [5], instead creating equal ground on which high quality research can be designed.

Over the past decade, co-research has become more prevalent across healthcare disciplines, including: palliative care services [6], community-based healthcare services [4] and transitions between healthcare services [7]. Within PEoLC, community-based participatory research has been called for to identify and address research gaps through collaboration between academic researchers and those with lived experience of an illness or their families and carers [47]. Co-research methodologies have been key in developing culturally sensitive palliative care interventions, bereavement drop in services, and pediatric palliative care [48–50]. Published recently, the Patients Changing Things Together (PATCHATT) programme supports individuals with a life-limiting illness to become involved in research with the aim of “leading a change that matters to them” [51]. Efforts have been made to produce guidance for involving individuals with lived experience of a condition in PEoLC research, that is sensitive to the additional ethical and practical considerations that must be taken with terminally ill individuals [52].

Evidence is emerging around the importance of co-research for engaging populations who may be experiencing exclusion from health care services, particularly when this emerges as a result of social, economic, and environmental barriers [8]. Recent research has adopted co-research methodologies with people from: minoritised ethnic groups [9], people experiencing homelessness [10], and those with substance use disorders [11, 12].

In the UK, the National Institute for Health Research (in partnership with INVOLVE) are the main body issuing guidance for co-producing research with communities [13]. They state five key principles for high quality co-research: “sharing of power; including all perspectives and skills; respecting and valuing the knowledge of all those working together on the research; reciprocity; building and maintaining relationships” [13]. Others also make recommendations to best practice for general co-production of general academic research [14]. Although this general guidance is undoubtedly helpful for co-producing some research, it is likely that particular care, consideration, and adjustments may be required when co-producing with inclusion health groups in PEoLC research.

The current paper considers four inclusion health groups as defined previously by Luchenski [12]: people with experience of homelessness, substance use disorder, incarceration or exchanging sex for money. The four inclusion health groups outlined experience severe health inequities with higher prevalence of chronic co-morbidities and high standardised mortality ratios that the general population and [17–19, 52]. Despite this disparity, they continue to underuse and have difficulty accessing palliative care services [20, 21]. If individuals from these inclusion health groups do access palliative care services, it is often very late in the development of their illness [21]. Some literature has been published regarding co-production of research with these populations. In one report, the authors critically reflected upon co-research methodologies with inclusion health groups, in turn creating a tool “to foster reflexivity in codesign” [8]. In Canada, an alliance of individuals with lived experience of homelessness hoped to promote equitable representation of people experiencing homelessness from service providers, researchers, and policy makers [15]. Consequently, they created seven principles for inclusion of people experiencing homelessness in research, stating “nothing about us, without us” [15]. Some guidance has also been published by organisations: Pathway, a leading homelessness charity in the UK, published a handbook to encourage individuals with lived experience of homelessness to become Experts by Experience [16]. This lays out expectations, boundaries, and responsibilities of both the researchers and experiential experts to foster safe and productive co-research [16]. Thus, some individual academics and organisations are beginning to involve lived experience co-researchers in research of varying fields.

During the development of this review, the authors intended to explore and synthesise studies of best-practice guidance for involving inclusion health groups as lived experience co-researchers in PEoLC research. However, we found that no such guidance existed (to the best of our knowledge). Consequently, the decision was made to synthesise findings from qualitative studies that discussed reflections of challenges and facilitators to involvement. In the UK, government healthcare policy states that applications for funding “must describe public involvement”, including active lived-experience co-research [22], yet there appears to be little explicit guidance, specific to this field, supporting applicants to do so. Excluding inclusion health groups from codesign processes may lead to only considering “dominant constructions of health and health care that may unintentionally reinforce oppression and existing inequities” [23].

### Aims and objectives

The aim of this review is to explore what the challenges, barriers, and facilitators are for involving people from four inclusion health groups as co-researchers in palliative and end of life care research. The four inclusion health groups considered are: people experiencing homelessness, substance use disorders, exchanging sex for money and incarceration. The objective of this review is as follows:

To identify and synthesise existing qualitative research outlining challenges, barriers, and facilitators, for involving inclusion health groups with lived experience, in palliative and end of life care research, from the perspectives of both the researchers and individuals with lived experience.

## Methods

Rapid systematic review methodology was used. As there is no universal definition of rapid review, we assumed a working definition: “a rapid review is a type of knowledge synthesis in which components of the systematic review process are simplified or omitted to produce information in a short period of time” [46]. However, principles for traditional systematic reviews (as outlined within the PRISMA 2020 checklist for systematic reviews) were adhered to: checkpoints on the PRISMA 2020 checklist were considered in preparing and carrying out this rapid review, including a traditional PRISMA flow-diagram to illustrate study selection [25]. This rapid review was undertaken using an approach to qualitative evidence synthesis (QES) called thematic synthesis [24]. In using a thematic synthesis approach, we aimed to provide a deeper understanding of the experiences of both academic and lived experience co-researchers involved in PEoLC research described throughout previous studies. The reporting of this review is guided by the ENTREQ statement [26].

### Searches

Keyword search strategies were devised within the research team prior to conducting searches: the full electronic search strategy can be seen in Table 1. Searches were conducted on 30/08/2022 on three electronic databases: PubMed, Medline and PsychINFO. These databases were chosen as they are large, medically relevant citation databases that were expected to identify eligible, topical papers. Additional grey literature was sought via internet searches (Google Scholar, OpenGrey), relevant charity and organisational websites, and expert recommendation.

**Table 1:** Keyword search strategy for electronic databases

Lived experience	(exp lived experience) OR (expert by experience) OR (exp substance abuse) OR (intravenous/) OR (exp substance related disorders) OR (exp vulnerable populations) OR (exp prison*) OR (exp homeless persons) OR (homelessness) OR (exp sex workers) OR (exp drug users) OR (exp prostitution)
(Research) involvement	(research involvement) OR co-research* OR (Patient and Public Involvement) OR PPI OR (research engagement) OR engaging OR (research participation) OR (collaboration)
Palliative care	(palliative care) OR palliative* OR (palliative medicine) OR (end of life) OR (end-of-life) OR EOL

### Eligibility criteria

The full eligibility criteria can be seen in Table 2 below. Studies eligible for inclusion were of any design, and described reflections, newer than 2012, guidance or recommendations for involvement of individuals with lived experience in PEoLC research. The chosen definition of inclusion health groups that forms the basis of lived experience was adapted from Luchenski [12]. This meant studies were eligible if they involved people with lived experience of homelessness, substance use disorder, incarceration [27], or sex work: four groups of individuals for whom literature has evidenced a high degree of intersection and low uptake of palliative care despite high need [28, 29]. The year 2012 was chosen as the lower limit for research publication to recognise the increase in importance of Patient and Public Involvement over recent years [30], whilst allowing inclusion of the most relevant, recent literature.

**Table 2:** Eligibility criteria for papers to be included in synthesis

Participants	To be eligible for inclusion in this review, studies must describe:· Adults (18+)· Lived experience (current or past) that is the subject of the individuals involvement in the research (i.e., homelessness, substance use disorder, incarceration, sex workers [12]· Involvement of individuals with lived experience is in an ‘informing the research’ capacity (e.g., advisory group/co-research), not a participant capacity· OR researchers reflections on involving people with lived experience in their work
Types of studies	· Original, peer-reviewed articles · Non-peer reviewed papers e.g., dissertations, grey literature, other non-peer reviewed guides · Papers conducted using any qualitative methodology
Outcomes	Any formal or informal exploration of best-practice methods, guidance, recommendations, barriers, or facilitators for involving individuals with lived experience in palliative and EOL research. This can be from the perspective of researchers, or lived experience (LE) contributors
Publication characteristics	· English language · 10 years (i.e., January 2012–August 2022)

### Selection and data collection

**Fig. 1:**
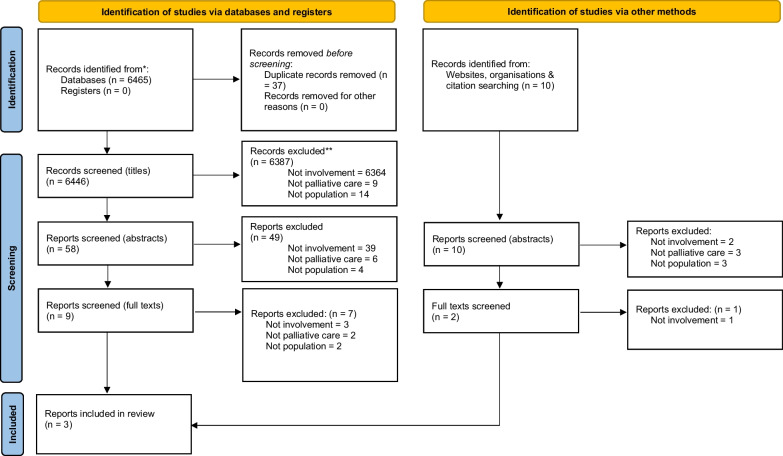
PRISMA flow-diagram of the screening process

Search outputs were imported into a reference management software (EndNote) and records deduplicated. One member of the research team (JC) screened all titles for inclusion/exclusion. A random 10% of titles were selected for independent second screening by another member of the research team (BH).Discrepancies were primarily resolved through discussion between JC and BH; if a third opinion was required, the wider research team was consulted. This approach was agreed upon to overcome time and resource constraints, while still encouraging measurement of consistency between reviewers [31]. In this case, there was 99.54% agreement in eligibility between researchers. Abstracts and full texts were subsequently screened via the same process. A breakdown of the screening process can be seen in Fig. 1.

Data were extracted from accepted full texts (n = 3). Data extraction was conducted manually by one member of the research team (JC) into a pre-designed form. This collected study data (including aims, design, and methodology), cohort data about the lived experience co-researchers (type of lived experience, current/past, number of contributors), and data pertaining to the outcome measures. In the eligible papers, reflections on the process of co-research were offered from the perspectives of both academic researchers without lived experience, and co-researchers with lived experience. Therefore, our extracted data are from both perspectives, including both first-order data (quotes from individuals with lived experience), and second-order data (original authors’ interpretations and reflections).

### Critical appraisal

The Joanna Briggs Institute Critical Appraisal checklist was used for critical appraisal of included studies [32]. All studies used the ‘Checklist for Qualitative Studies’. Critical appraisal was completed by one member of the research team (JC). Papers were scored as high (90–100%), medium (80–90%) or low (70–80%). Results of the critical appraisal can be seen in Table 3.

**Table 3:** Characteristics of included studies

Author Year	Summary aims	No. lived experience contributors	Lived experience type	Type of guidance	JBI Critical Appraisal Score
Visser 2021 [34]	“To encourage others to harness the benefits of co-producing research with people with lived experience of health care in prison”	3	Lived experience of cancer care in prison	Reflections and guidance	85% (Medium)
Abrahams 2015 [36]	“To explore the ethical and practical dilemmas which arise when including service user advisors within a research team.”	3	Homelessness	Reflections	75% (Low)
Penrod 2016 [35]	“To illustrate key methodological strategies in the application of PAR methods in a study designed to promote sustainable insider-generated system-level changes in the provision of end-of-life (EOL) care in the restrictive setting of state prisons”	18	Providing palliative and end of life care in prison settings (staff)	Reflections	70% (Low)

### Analysis and synthesis methods

Analysis followed thematic synthesis methodology described by Thomas and Harden [24]. This was selected to account for the variety of qualitative methodologies expected to arise within individual papers; to explore similarities, differences and relationships across studies while aiming to provide novel insights and interpretations, above and beyond the studies individually [24, 33]. The synthesis followed a three-step process: (1) line by line coding of the text, (2) development of descriptive themes, and (3) generation of analytical themes.

Extracted data was imported in NVivo™, a qualitative analysis software. Line by line coding of the data was carried out by one member of the research team (JC). An inductive approach was selected to enable context to be considered in individual papers: for example, where reports offered differing interpretations of whether a component was a challenge or facilitator. Coded data from each paper were allocated into existing codes from the first paper, or additional codes were created depending on relevance and fit. Codes were then considered collectively, and initial descriptive themes were developed (JC). These descriptive themes were discussed with the wider research team (BH, CS, KF) and further analytical themes developed. Regarding reflexivity, all members of the research team come from a range of backgrounds including psychology, medicine, palliative care nursing, mixed methods systematic reviewing, and psychiatry. The synthesis was also presented to and discussed with a wider steering group of professionals: this was a pre-existing steering group for the larger programme of work, consisting of a range of professionals across palliative care, psychiatry, homelessness, addiction services and hostel staff. One member of the steering group also had lived experience of homelessness.

## Results

### Study selection

Electronic database searches identified 6446 papers after de-duplication. After completion of the screening process outlined earlier, three papers remained eligible for inclusion in the analysis [34–36]. The full study selection process can be seen in Fig. 1. Characteristics of included studies can be seen in Table 3: study quality ratings from the Joanna Briggs Institute critical appraisals can also be seen in Table 3.

## Findings

### Description of included studies

Of the three included papers, two involved individuals with lived experience of incarceration: experience of cancer care in prison [34], and experience of providing palliative and end of life care in prison settings [35]. The latter used professionals and prison-staff as a proxy for prisoner views and experiences. The third report involved women with previous lived experience of homelessness [36]. Specifically, the lived experience voices represented from individual studies within the current review are: two women and one man with lived experience of cancer care in prison-settings [34]; two women with lived experience of homelessness (referred to as ex-service users) [36]; and eighteen professionals responsible for provision of end of life care in prison-settings (used as a proxy of inmates themselves due to concerns over wellbeing and emotional burden) [35]. There were no papers offering reflections or guidance on involving individuals with lived experience of sex work or substance use disorder in palliative and end of life care research (Table 4).

**Table 4:** Themes within thematic synthesis and the perspectives that were pertinent to each theme

Theme	Sub-theme	Perspective included
Challenges & Barriers	How lived experience impacts positionality: bias and scepticism	Lived experience co-researchers
	Protection vs. patronisation	Academic researchers
	Overcoming stigma and reluctance to share power	Academic researchers
	Emotional burden of research	Both
	Facilitating appropriate reimbursement	Both
Successes & Benefits	Relatability of lived experience co-researchers	Lived experience co-researchers
	Advanced level of insight	Academic researchers
	Two-way learning opportunity	Both
Advice & Guidance	Promoting equal relationships	Academic researchers
	Continued support	Both
	Allocation of sufficient funding	Academic researchers
	Tailored, accessible training	Both

All three papers offered reflection-based feedback, with some general advice. Two papers [34, 36] utilised reflections from both the lived experience co-researchers and the academic researchers. The third offered general reflections from the perspective of the research team, although no quotes or individualised feedback was included. No papers offered formalised guidance for involving those with lived experience in palliative and end of life care research.

Only one paper reported involving lived experience co-researchers throughout the entire research process [34] including: conceptualisation, development of study materials, involvement in data collection via conducting interviews (with an academic researcher), and data analysis. The other two papers utilised lived experience co-researchers in an advisory manner; they were consulted regarding study materials and procedures, but their involvement did not extend to data collection or analysis [35, 36].

Each paper recruited lived experience co-researchers differently, via partnering with a third-party organisation [34], utilising existing connections from previous projects [36], or structured, targeted recruitment within a pre-defined group of individuals [35]. Post-recruitment, all papers reported some form of training and preparation for their lived experience co-researchers. Topics of training included background and theory to the research topic, basic research methods and interview skills. Emphasis was placed upon the importance of tailoring training to the individual to avoid patronisation [34]. Considering accessibility of training was also deemed essential, particularly regarding expenses such as transport and catering [35, 36].

### Findings arising from the thematic synthesis

The synthesis identified three key themes which illustrate the challenges and successes of involving inclusion health groups with lived experience in PEoLC care research. Each theme portrays the different perspectives of those involved i.e., academic and lived experience co-researchers. Each key theme is further divided into sub-themes.

### Challenges and barriers

#### How lived experience impacts positionality: bias and scepticism

One challenge appeared unique to lived experience co-researchers: co-researchers reflected on how their previous experiences of a situation impacts their perceptions of it happening to others. Individuals with lived experience of incarceration, and experiences of advanced disease and palliative care within prison, expressed that it was difficult to remain unbiased in their new role as a member of the research team when facing participants or conducting analyses; they had pre-conceived thoughts and opinions around the situation. In one study where lived experience co-researchers were involved in interviewing prison staff, pre-existing scepticism and lack of trust in prison staff led lived experience co-researchers to approach the interviews with a negative mindset: “*I imagine prison officers wouldn’t even believe a prisoner had cancer if they told them themselves, not until they saw medical evidence”.*[34, page 8] This was also evident in situations where participants recounted experiences that differed from the lived experience co-researchers own.

“SW’s personal experience of healthcare, and that of her friends, in prison was predominantly negative, and the limited access to pain relief in prison awakened a need for activism. This meant SW approached interviews in which patients in prison recounted quite positive care experiences with a level of scepticism and cynicism”. [34, page 4].

### Protection versus patronisation

An important challenge for researchers, was a fear of involvement appearing tokenistic. They struggled to balance allowing co-researchers full involvement to promote equal relationships, with recognising when to limit co-researcher involvement to avoid harm (for example, exposure to distressing, or potentially re-traumatising situations). Key to this theme, therefore, is that academic researchers considered these inclusion health groups as needing protection, thereby being somehow ‘vulnerable’. Within this, the academic researchers feared that decisions they made intending to protect co-researchers would be received as patronising.

However, when one study explicitly asked lived experience co-researchers whether they had felt patronised, they responded: “N*o I’ve never felt patronised or kind of … you’ve always been inclusive, you know. And I’ve got no doubt in my mind that maybe you’ve had to be mindful of your vocabulary for instance and not … and all them things don’t go unnoticed”. *[36, page 20] While academic researchers were concerned that their well-intentioned protection would be received as patronisation, lived-experience co-researchers were able to dismiss this concern. Lived experience co-researchers recognised and appreciated the academics’ efforts to promote equal relationships whilst being mindful of potential harm.

#### Overcoming stigma and reluctance to share power

Some researchers reflected upon the resistance they faced by other researchers when proposing and organising involvement of lived experience co-researchers. In one case, the principal investigator and research assistant advocated for lived experience involvement, but met hesitancy from the wider research team and advisors. One specific concern, in a study where lived experience co-researchers were service users of a women’s homelessness service, was confidentiality.

This averseness occurred despite knowledge of the theory behind, and benefits of, involving lived experience co-researchers in research. When academic researchers reflected on why others may be hesitant to work alongside lived experience co-researchers, they suggested that this may be due to pre-existing prejudices and internal struggles relating to power sharing and equality. This was reflected to occur despite knowledge of power-sharing as best practice.

“This attitude towards service users, can, perhaps, be seen as symptomatic of the reluctance, in practice, to share power, even by those who accept this in theory”. [36, page 20].

#### Emotional burden of research

One key challenge that was reflected upon by both researchers and lived experience co-researchers was the emotional strain of lived experience involvement in palliative and end of life care research. This emotionality impacted researchers and lived experience co-researchers in different ways. Academics reflected that facilitating research involving ‘vulnerable’ groups with lived experience added an additional layer of emotional labour to already emotion-provoking research topics. Researchers felt responsible for the emotional wellbeing of lived experience co-researchers, additional to ensuring their own welfare.

“On interview days I was not only worried for my own feelings but also worried about the responses of lived experience researchers to the environment. Looking back, I feel it is a weird mix of feeling responsible for someone else, and feeling you, for some reason, should be able to cope with things better because you are an “academic”.”[34, page 5].

For one study, this consideration of emotional burden on co-researchers led to the decision to limit involvement to an advisory role, removing opportunities for lived experience co-researchers to actually conduct the research [36]. How academic researchers made this decision on behalf of co-researchers, although well-intentioned to ‘protect’ them, removes any equity in the relationship, instead positioning the academic researchers as the lead decision makers.

Throughout some research, lived experience co-researchers were sometimes asked to re-visit (either physically or mentally) stressful situations in which they had once lived. This included returning to a prison in which they had spent years of their lives, to conduct research interviews [34]; or reflecting upon their experience of advanced ill-health during homelessness [36]. Reflections from lived experience co-researchers in each study highlights how this exposure to distress inducing triggers can impact their emotional wellbeing.

“On the way to the prison to meet the other researcher [RV], I felt mixed emotions; excitement, apprehension and fear. I walked alongside the huge enclosing prison wall when I had to take a moment and gather my thoughts.”[34, page 5].

Examples of this distress were provided by lived experience co-researchers. Specific worries were held around being denied access to a prison setting, or being “kept in” once there: “*I was thinking that [researcher] was naïve, that I would be let into the prison in the first place, I thought they would be looking for an excuse to knock me back”*. [34 page 5] These emotional reactions were also experienced without physical exposure to the environment, through reading others accounts: *“Whilst reading one transcript, I found myself physically triggered and the side effects of my injection hit me quite hard and I had to stop and lay down*”. [34, page 7].

#### Facilitating appropriate reimbursement

Whilst academics involved in all three papers agreed that lived experience co-researchers should be reimbursed, there were conflicting opinions regarding best practice. Two out of three papers cited INVOLVE [37], who offer guidance applicable to general public involvement in research. Aligning with the general co-research principle of fostering equal, collaborative relationships that are free of power dynamics, financial reimbursement illustrates a “*tangible acknowledgement of [Expert By Experience] involvement*” [36]. Nonetheless, academics’ reflections described concerns about the impact of offering monetary incentives: “*monetary incentives may attract those in urgent need of cash, but who have no intention of staying with the project long term*”. [36, page 19] This theme links to the aforementioned stigma surrounding inclusion health groups: academics’ reluctance to offer fair payment illustrates their hesitations around powerless relationships in which all members of the team are equal.

Underlying this debate were differences in how researchers perceived lived experience contributors; whether they were research-specific advisors with invaluable experience, or service users providing feedback who typically may not receive payment. One report importantly asked for input from lived experience co-researchers on this challenge, who emphasised how “*we were now in a different relationship; and in the new situation, vouchers could be seen as being patronising and lacking in trust*”. [36, page 19] However, this reflection was only asked for after completion of the project—the lived experience co-researcher had no input on payment method throughout the project. This illustrates how academic-lived experience relationships may never be truly powerless, so long as important decisions around payment are made solely by academics’, without consideration of what is most important for lived experience researchers.

### Successes and benefits

Three main success and benefits of involving lived experience co-researchers in PEoLC research were identified.

#### Relatability of lived experience co-researchers

Presence of lived experience researchers can be a great equaliser within research activities. This benefit was recognised within reflections provided by lived experience contributors across the studies. Particularly when involved in participant facing activities, their involvement promoted trust and feelings of safety; particularly important for vulnerable groups such as those involved here. Lived experience co-researchers understood jargon, built rapport and connections, and quickly encouraged meaningful discussion with research participants: “*AX suggests it was a real benefit that he was one of the researchers, as he was familiar with prison jargon: “Never mind the accents, maybe the interviewees were feeling more confident speaking to me—for someone to relate to—you (RV) might have missed some things if I wasn’t there””*. [34, page 6] Building trust quickly was particularly important when in prison-settings, as interviews were sometimes unexpectedly cut short as people returned to cells for safety concerns.

#### Advanced level of insight

Researchers reflected upon the ability of lived experience co-researchers to offer insight that otherwise may not have been achieved. This was evidenced during analysis of interview transcripts, as contributors used their lived experience to offer deeper understanding of the content within and language used throughout the transcripts. Although previously mentioned as a challenge, some researchers reflected that lived experience co-researchers scepticism was a benefit, as it encouraged researchers to challenge their initial perceptions of transcript content [34]. The differing positionality of academic and lived experience co-researchers can result in a more complete understanding and analysis. Lived experience co-researchers insight was also valuable for advanced practical knowledge around carrying out research that academic researchers may struggle to access: “*Examples of contextual demands included the extent to which special dispensation was granted to permit inmate visits in the infirmary, the extent of inmate interaction with each other and with staff, and the differences in physical structure of work units*”.[35, page 12].

#### Two-way learning opportunity

The most frequently coded theme prevalent in all three papers, across academic and lived experience researchers expressed how lived experience involvement in research facilitates a mutually beneficial learning opportunity. Academic researchers reflected upon how presence of lived experience co-researchers in emotionally challenging scenarios provided a source of reciprocated support: *“As prison is such a strange research environment, I was relieved that I did not conduct the interviews on my own and shared this experience with lived experience researchers”*. [34, page 5] Furthermore, academic researchers could gain experience and confidence in conducting inclusive, co-operative research.

Concurrently, lived experience researchers benefitted from involvement, too. Involvement allowed them to create new, positive lived experiences – a potentially therapeutic, cathartic exercise that serves to remove some perceptions of power dynamics between themselves and authority figures in past situations. One lived experience co-researcher stated: *“Going into the female prison that I had spent time in as a prisoner, however, did have an impact on me and I think, probably, in a good way. I was able to exorcise some demons surrounding the prison officers I had known previously”.* [34, page 7] This illustrates how there are other power relationships to consider for inclusion health groups, beyond those between academic and lived experience researchers.

For lived experience co-researchers, involvement in research generated feelings of giving back and helping others.

“For Joe and Lucy, it was apparent that being a full part of the project had enabled them to put feelings previously in the background into words and ideas about the best way to work with women and also the confidence and ability to take action and challenge decisions effectively.”. [36, page 22].

### Advice and guidance

The synthesis identified four areas relating to providing advice for researchers considering involvement of co-researchers with lived-experience.

#### Promoting equal relationships

The most frequently cited advice (from the perspective of both academic and lived experience co-researchers) was to dedicate time to promote truly collaborative, equal relationships between academic and lived experience researchers.

“Collaborative relationships between academic and lived experience researchers only work when there is a commitment to investing time and energy in the building of relationships. And this is something that takes time and cannot be rushed”. [34, page 10].

This means acknowledging, addressing, and removing power dynamics. For some, this involved allowing lived experience co-researchers to take the lead where appropriate. Places in the reviewed reports that could have arguably better considered power sharing were determining method and timing of payment and extent to which a co-researcher with lived experience needing ‘protecting’. Relationships should be openly discussed and agreed upon prior to commencing a project, so all expectations are managed. Fostering equal standings for everyone is particularly important for those with lived experience of incarceration, as enforcing power dynamics may be traumatic or stressful.

#### Continued support

Linking to the potential for emotional distress mentioned earlier, both academic and lived experience researchers were aware of lasting emotional effects of sensitive research. Advice suggests that continued debriefs should be offered throughout involvement (i.e., after each stage of involvement), as well as check-ins for a period after the end of the project.

“A year has elapsed since the first interview, but when discussing the interviews it can feel like they happened yesterday. It is therefore advisable to have ‘check-ins’ with lived experience researchers on a regular basis, even after the fieldwork part of research has finished.” [34, page 7].

#### Allocation of sufficient funding

Budgeting for lived experience involvement should be done from an early stage; as early as study conception and funding application. In order to do this, decisions must be made early in the process about how lived experience researchers will be reimbursed for their time. Papers included in the current review suggest, if possible, applying for and allocating more than is anticipated to be needed. This can help to avoid situations in which the research would benefit from lived experience co-researchers input, but funds have been exhausted (perhaps at a later stage of the project).

#### Tailored, accessible training

Training was deemed essential for involvement. However, any training should be tailored to existing knowledge to maximise productivity and avoid patronisation. This is particularly important in fostering equal relationships – training could focus on drawing out the differing expertise of both academic and lived experience contributors. Papers included in the current review also discussed the importance of accessibility of training, to avoid additional strain on lived experience co-researchers.

“So like silly little things that make a difference, you know, paying parking and finding parking … and you underestimate just them silly little things, how important they are … to support people in coming to meetings, making sure there’s lunch. All them little things help the engagement of … specially people like us service users.” [36, page 18].

## Discussion

This rapid review explores existing literature outlining barriers, facilitators, and benefits for involving inclusion health groups with lived experience in palliative and end of life care research. No papers were found to offer formal, best practice guidance for involving inclusion health groups in PEoLC research. Only three papers were identified as eligible for inclusion in this review. Included papers involved co-researchers with lived experience of incarceration (2/3 papers), or homelessness (1/3 papers) – none involved those with substance use disorder or exchanging sex for money in palliative and end of life care research. Thematic synthesis of qualitative studies was conducted using reflections from the perspectives of both academic and lived experience researchers. Identified challenges included: facilitating appropriate reimbursement; overcoming stigma and reluctance to share power; protection vs. patronisation; how lived experience impacts positionality and the emotional burden of research. Successes and benefits included: advanced level of insight, a two-way learning opportunity and relatability of lived experience co-researchers.

To the authors best knowledge, this is the first review to look at people’s experiences of being lived-experience co-researchers, or involving inclusion health groups as lived experience co-researchers in the field of palliative and end of life care. There is little guidance on involvement in PEoLC research compared to other fields [38, 39], and less still when considering involvement of marginalised groups (as opposed to patients, carers, or others more generally).Given the impact of exclusion on health, the young age at which people who have been multiply excluded experience health issues associated with older age in the general population, and the often currently missed opportunities for palliative care support, this is a gap that urgently needs to be addressed.

Nonetheless, some current findings are akin to similar previous studies with people from the general population. The finding that lived experience co-researchers personally benefited (via self-growth and upskilling) from research involvement appears consistent across literature and areas of research [40, 41]. This is particularly strongly highlighted in a recent systematic review considering co-research with patients from the general population who were concurrently experiencing cancer specific care with palliative care [42]: they found considerable emotional, cognitive, and social benefits to the lived experience co-researchers, including acquiring new skills, self-growth and increased confidence, and a feeling of ‘giving back’. The potential for an emotionally distressing yet cathartic experience for vulnerable groups within co-research has also been recognised: older adult co-researchers with experience of homelessness reflected that “telling their stories was both difficult and therapeutic” [43].

One important yet complex point is around power relationships in co-research, particularly with inclusion health groups. In the three included reports, academic researchers viewed co-researchers as needing protection; as ‘vulnerable’. There are undoubtedly sensitivities with carrying out co-research around topics of PEoLC with these groups, such as potential for re-traumatisation or exposure to potentially triggering situations. However, positioning academic researchers as ‘protectors’ of ‘vulnerable’ lived experience co-researchers, inherently forms a power-based dynamic. This power relationship is further exacerbated by some practical decisions falling solely on academics, such as payment method or timing. This may be expected, as academics have to offer payment that is in line with, and approved by their institutions. However, this dynamic places the academic researchers as ‘leaders’, above lived experience co-researchers. The true extent and complexity of power relationships in co-research, especially when considering lived experience co-researchers is vast, and perhaps we are unable to fully untangle this. However, it is something about which we should be mindful when formulating and carrying out co-research.

### Involving the voices of Experts by Experience

Building upon the rationale for including lived experience co-researchers in research initially, it seems inappropriate and insufficient to attempt to create guidance for a group of individuals without consulting them and considering their perspectives. Importantly, some themes within the current review were generated using reflections made by individuals with lived experience within the original papers. The lived experience voices represented from individual studies within the current review are: two women and one man with lived experience of cancer care in prison-settings [34]; two women with lived experience of homelessness (referred to as ex-service users) [36]; and eighteen professionals responsible for provision of end of life care in prison-settings (used as a proxy of inmates themselves due to concerns over wellbeing and emotional burden) [35]. This provides huge strength to the review, yet the paucity of research involving people with lived and experience, and limited reflections from all involved highlights a need for additional exploration and indicates that involvement in research may currently be the exception rather than the norm.

### Limitations

The current report is a rapid review conducted over 4-months. Rapid reviews may be considered less comprehensive than conducting a full systematic review. However using a rapid review method was a purposeful decision to combat time restraints and to identify practical recommendations from existing literature to help shape an ongoing project. Despite this, care has been taken to ensure rigour, including: independent second screening at all stages of search output (title, abstract and full texts), critical appraisal methodologies to assess risk of bias and study quality, and adhering to transparent reporting guidelines (ENTREQ). In addition, experts within the field of palliative care, inclusion health and homelessness peer advocacy were consulted in the identification of relevant literature. Nonetheless, the hastened nature of this rapid review may have resulted in some relevant papers or abstracts being missed.

The eligibility criteria applied ensured only studies involving the four identified inclusion health groups were included. This purposeful decision was taken to maintain a focused scope within this review. There are undoubtedly other inclusion health groups for whom involvement in PEOLC research is essential. Included studies were also within the field of palliative and end of life care. This led to exclusion of two studies that presented guidance for involving vulnerable groups as lived experience researchers in other fields of research [44, 45]. When developing guidance for involvement of inclusion health groups within PEoLC care research, perhaps these similar studies could be consulted to develop initial understanding.

We did not involve individuals with lived experience of inclusion health and exclusion in developing and conducting this review. However, this review will form the first step of the authors wider work: understanding the current research landscape and what (little) guidance exists through the current review will inform the authors upcoming qualitative work to develop a best practice guide for involving people with lived experience of homelessness in palliative and end of life care research. This consequent qualitative research will heavily involve individuals with experience of homelessness, in partnership with a leading UK homelessness peer advocacy organisation.

## Conclusions

To the authors best knowledge, no published papers have produced formal, best practice guidance for the involvement of inclusion health groups in PEoLC research. Few qualitative studies have reflected on the challenges, barriers, and facilitators for involving co-researchers with lived experience of homelessness or incarceration in palliative and end of life care research: none considered involvement of individuals with experience of substance use disorder or exchanging money for sex. This paper highlights some of the potential barriers or facilitators to involvement that are important to co-research with inclusion health groups who are otherwise under-represented or excluded communities. There is a need for comprehensive, formal research about co-research best practice with inclusion health groups in palliative and end of life care to inform future policy recommendations and ensure the safety and impact of future co-produced research.

### Recommendations

*Further research into facilitating involvement of inclusion health groups with lived experiences in palliative and end of life care research:* Future research could offer a detailed consideration and exploration of one inclusion health group with severe social exclusion (i.e., experience of homelessness, incarceration, substance misuse, and sex work) to provide detailed understanding on how best to facilitate lived experience involvement in palliative and end of life care research. Future research should include the voices of both academic researchers and individuals with lived experience to promote the co-research ethos of “nothing about us, without us” [15].

*Development of (formal) guidance or policy for facilitating involvement of inclusion health groups with lived experiences in palliative and end of life care research:* Written guidance and recommendations for safe, inclusive, and impactful involvement of inclusion health groups in PEOLC research is needed. This guidance should consider the complexities specific to carrying out PEoLC co-research with inclusion health groups. This guidance could be aimed at academic researchers, policy developers, or anyone wishing to involve individuals with lived experience in their palliative and end of life care projects. Lived experience co-researchers should be consulted in the creation of this guidance.

## Supplementary Information

**Additional file1**. Use of ENTREQ reporting criteria for this rapid review.

## Data Availability

All data relevant to the study are included in the article or can be accessed via contacting the corresponding author (Jodie Crooks: jodie.crooks@mariecurie.org.uk).

## Abbreviations

PEoLC: Palliative and end of life care
QES: Qualitative evidence synthesis
ENTREQ: Enhancing transparency in reporting the synthesis of qualitative research
JBI: Joanna Briggs Institute
